# Stem Cells: Present Understanding and Prospects for Regenerative Dentistry

**DOI:** 10.3390/jfb15100308

**Published:** 2024-10-15

**Authors:** Angelo Michele Inchingolo, Alessio Danilo Inchingolo, Paola Nardelli, Giulia Latini, Irma Trilli, Laura Ferrante, Giuseppina Malcangi, Andrea Palermo, Francesco Inchingolo, Gianna Dipalma

**Affiliations:** 1 Department of Interdisciplinary Medicine, University of Bari “Aldo Moro”, 70124 Bari, Italy; angeloinchingolo@gmail.com (A.M.I.); ad.inchingolo@libero.it (A.D.I.); drnardellipaola@gmail.com (P.N.); dr.giulialatini@gmail.com (G.L.); trilliirma@gmail.com (I.T.); lauraferrante79@virgilio.it (L.F.); giannadipalma@tiscali.it (G.D.); 2 College of Medicine and Dentistry, Birmingham B4 6BN, UK; andrea.palermo2004@libero.it

**Keywords:** stem cells, oral tissue regeneration, regenerative dentistry

## Abstract

Regenerative medicine in dentistry focuses on repairing damaged oral tissues using advanced tools like stem cells, biomaterials, and tissue engineering (TE). Mesenchymal stem cells (MSCs) from dental sources, such as dental pulp and periodontal ligament, show significant potential for tissue regeneration due to their proliferative and differentiative abilities. This systematic review, following PRISMA guidelines, evaluated fifteen studies and identified effective strategies for improving dental, periodontal, and bone tissue regeneration through scaffolds, secretomes, and bioengineering methods. Key advancements include the use of dental pulp stem cells (DPSCs) and periodontal ligament stem cells (PDLSCs) to boost cell viability and manage inflammation. Additionally, pharmacological agents like matrine and surface modifications on biomaterials improve stem cell adhesion and promote osteogenic differentiation. By integrating these approaches, regenerative medicine and TE can optimize dental therapies and enhance patient outcomes. This review highlights the potential and challenges in this field, providing a critical assessment of current research and future directions.

## 1. Introduction

The hard and soft tissues of the oral cavity (teeth, periodontium, jaw bones, salivary glands) form a complex multiorgan system that is closely interrelated in function and development [1,2,3]. Any damage to one of these structures (dental caries, periodontal disease, jaw bone atrophy, altered glandular function) can lead to functional and structural changes in the other tissues as well, with overall impairment of oral health [4,5,6].

The incidence of oral diseases is increasing as life expectancy increases [7,8,9].

Tissue engineering (TE) is an interdisciplinary field that combines principles of biology, engineering, and material science to develop biological substitutes that restore, maintain, or improve tissue function [10,11,12,13,14]. It primarily focuses on three key elements: stem cells, biocompatible scaffolds, and bioactive molecules such as growth factors or drug delivery systems. In regenerative dentistry, TE is aimed at developing materials with high regenerative potential to repair or regenerate damaged oral tissues [15,16,17,18,19,20].

Stem cells are undifferentiated cells that can self-renew and differentiate into specialized cell types, making them essential for regenerative medicine. They include embryonic stem cells (ESCs), which can become any cell type, and mesenchymal stem cells (MSCs), which can differentiate into tissues like bone, cartilage, and muscle. MSCs, particularly valued for their ability to modulate the immune system and promote tissue repair, are key in treating inflammatory and degenerative conditions [16,21,22].

**Figure 1:**
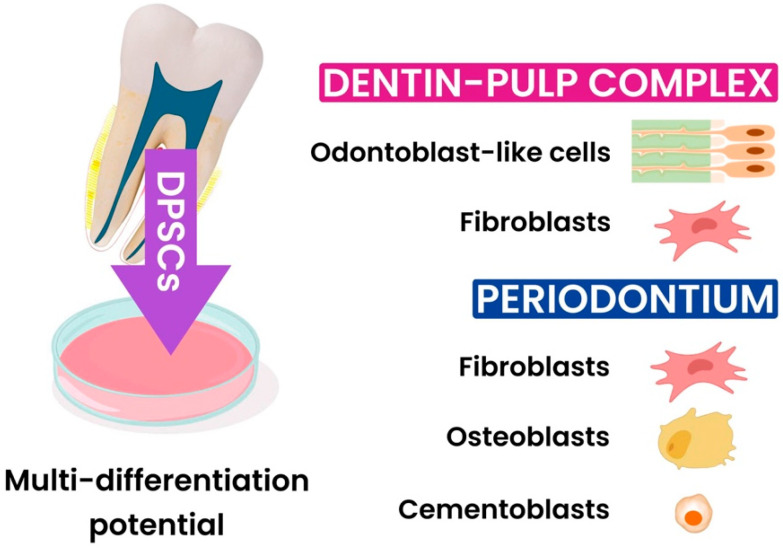
Dental pulp stem cells have the capacity to regenerate in various ways. Diagram illustrating the dental pulp stem cells’ capacity for multi-differentiation in the regeneration of periodontal and dentin–pulp complex tissues.

In oral and maxillofacial TE, MSCs like dental pulp stem cells (DPSCs), periodontal ligament stem cells (PDLSCs), and bone marrow mesenchymal stem cells (BMSCs) are being extensively studied for their potential to promote dental and bone tissue regeneration [23,24,25,26,27]. DPSCs, which originate from the neural crest, show significant osteogenic and dentinogenic potential. Their high proliferative capacity and ability to differentiate into multiple cell types make them ideal candidates for autologous regenerative therapies [5,28,29,30]. DPSCs have been explored for the regeneration of various dental structures, including the dentin–pulp complex and periodontal tissues [31,32,33] (Figure 1). The relevant studies were mainly based on the use of scaffolds and DPSCs derived from healthy pulp [34,35,36,37].

The relevant studies were mainly based on the use of scaffolds and DPSCs derived from healthy pulp [34,35,36,37].

For periodontal tissue regeneration, there are more clinical and preclinical studies. DPSCs extracted from the healthy and inflamed pulp and their secretome (present in the extracellular space and rich in bioactive molecules: growth factors, cytokines, chemokines, exosomes) have been used [38,39,40,41,42].

Recent studies have favored the use of secretomes consisting of cell extracts because they show better results, are easier to obtain, are more quantifiable, and are more stable over time [43,44,45,46]. As a cell-free product, the secretome can be more easily cataloged and characterized [47,48,49,50].

In TE of the salivary glands, only the submandibular gland and its secretome derived from healthy pulp have been used as the only model in preclinical studies with dental pulp stem cells (DPSC) [51,52].

For TE of cranio-maxillofacial bone defects, there are both preclinical and clinical studies using DPSCs derived from healthy pulp only [5,53].

The analysis of the scientific literature on the use of MSCs in TE of dental and maxillofacial tissues represents a multifaceted aspect, encompassing biochemical, biomechanical, molecular process imputation, and small molecule therapeutic processes. Each analysis presents different and tailored strategies with the goal of enhancing the therapeutic capabilities of MSCs by improving their regenerative effect and clinical applications [7,54,55,56].

Analysis of substances such as 2,3,5,4′-tetrahydroxystilbene-2-O-β-glucoside (THSG) and GSK-3 inhibitors have shown the ability to promote the proliferation and viability of DPSCs with beneficial therapeutic effects [57,58,59,60].

The biomechanics influenced by the presence of substrates of different elasticities (fibronectin-coated polydimethylsiloxane substrates of different elasticities) would direct the differentiation and proliferation of DPSCs into specific tissue types.

A study has shown how hydrogel (methacrylate hyaluronic acid (MeHA)) scaffolds are able to maintain the viability of epithelial and mesenchymal cells by promoting the signaling between them that is essential for cell proliferation. Thus, hydrogel scaffolds have been shown to be a promising bioengineering system for human tooth germ in vitro [61,62,63].

In the treatment of periodontal defects, the use of DPSCs results in bone regeneration due to both increased superoxide dismutase (SOD) and decreased IL1β levels [64].

Studies on PDLSCs have shown how prolactin-induced protein (PIP) and progranulin (PGRN) promote osteogenic differentiation, resulting in improved alveolar bone and dentin regeneration outcomes [65,66,67].

PDLSCs significantly reduce polymorphonuclear neutrophil (PMN) apoptosis and enhance antimicrobial activity. Lipoprotein A4 (LXA4) has been seen to offer new therapeutic options for the treatment of periodontitis, as it would enhance the potential of PDLSCs in regulating dental inflammation and improving tissue elasticity [68].

In both in vitro and in vivo studies of bone regeneration using rat bone marrow mesenchymal stem cells (BMSCs), it was observed that treatment with matrine (an alkaloid derived from the dried leaves of a leguminous plant, *Sophora flavescens*, from China) significantly enhanced osteogenic differentiation, as evidenced by increased alkaline phosphatase (ALP) activity and mineralization of BMSCs [69,70,71,72,73].

Studies on sandblasted and acid-etched titanium substrates have demonstrated improved adhesion, spreading, and osteogenic differentiation of induced pluripotent stem cells (iPScs) derived from human gingival fibroblasts. This highlights the important role of bioengineering in optimizing biomaterials to modulate stem cell behavior in craniofacial regenerative medicine [74,75,76,77,78,79].

The purpose of our research is to evaluate how TE using MSCs can be a challenge in generating oral tissues (teeth, periodontium, alveolar bone). Controlling the differentiation of DPSCs into specific cell lines is still a big question.

Integrating different results with synergistic approaches could further refine regenerative therapies in dentistry and oral medicine with MSCs and improve patients’ quality of life.

## 2. Materials and Methods

In this research, we used the PubMed, Web of Sciences (WOS), and Scopus databases using PROSPERO code ID CDR 565515 [80] to conduct a systematic review in accordance with the Preferred Reporting Items for Systematic Reviews and Meta-Analyses (PRISMA) criteria. We searched articles from the last ten years using the following keywords: “stem cells” and (“oral tissue regeneration” OR “regenerative dentistry”). The topic related to the most recent advancements and useful applications of regenerative dentistry techniques was prioritized, with a focus on the regeneration of periodontal, dental pulp, and alveolar bone tissue. To help the reader, we have supplied a clear research topic that asks, “How can biotechnology and engineering strategies improve dental pulp regeneration through the use of stem cells and growth factors?”. The selected papers were all full-text publications that discussed the application of stem cells both in vivo and in vitro. Moreover, all literature reviews, whether or not they included a meta-analysis, were disqualified. English-language articles were chosen, and on 18 March 2024, a final search was carried out. We carefully went through the provided literature’s references to find any further publications that might be pertinent.

The article extraction was carried out by two writers (F.I. and G.L.) in accordance with the inclusion criteria, and any disagreements were settled after sufficient discussion and comparison.

### Quality Assessment

The ROBINS-I technique was used by two reviewers, F.P. and P.A., to assess the quality of the included papers. ROBINS-I was developed to assess the potential for bias in the results of non-randomized studies assessing the effects of two or more medicines on health. A bias degree was assigned to each of the seven assessed points. In the event of a disagreement, F.I., the third reviewer, was consulted until an agreement was reached. The reviewers evaluated the possibility of bias in seven domains: confounding, participant selection, intervention classification, deviations from intended interventions, missing data, outcome measurement, and choice of reported results. They were trained to use the ROBINS-I tool and followed the guidelines. To increase the uniformity and objectivity of the assessments, disagreements or conflicts among the reviewers were resolved by discussion and consensus. In cases where a third reviewer was unable to achieve a consensus, that decision was definitive. The use of ROBINS-E for bias assessment allowed for a thorough evaluation of potential biases in the non-randomized trials that were part of this investigation. By highlighting the benefits and drawbacks of the evidence base, it enhanced the assessment of the overall quality and reliability of the findings. By considering the possibility of bias, the authors of this review were able to arrive at more knowledgeable interpretations and findings based on the available data.

## 3. Results

**Figure 2:**
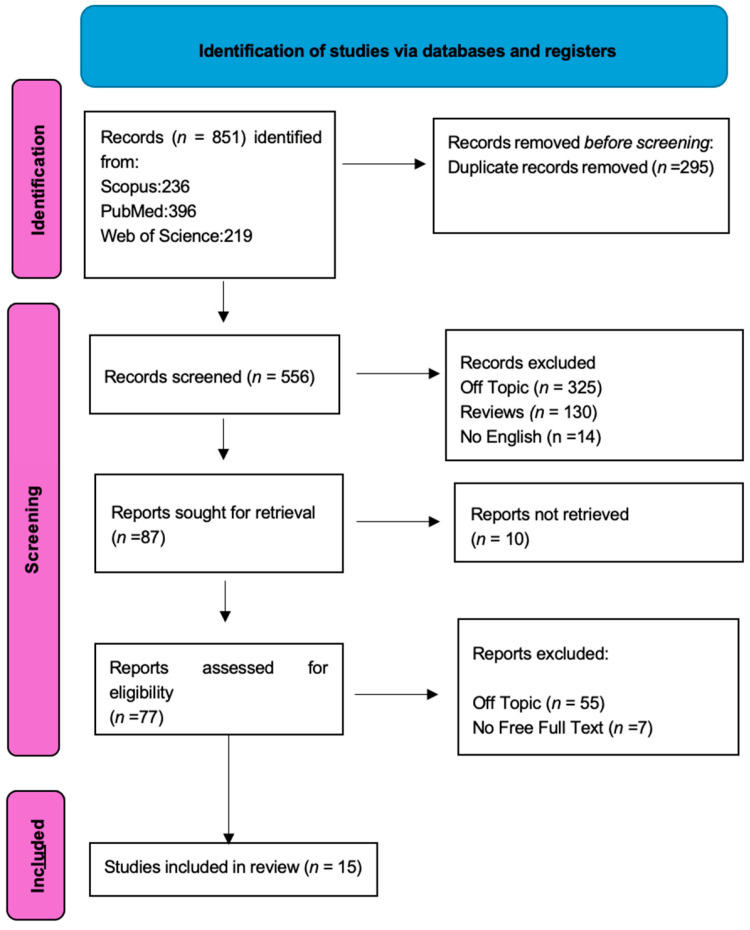
PRISMA flowchart following guidelines.

Initially, a total of 851 documents were identified through the literature search: 396 from PubMed, 219 from Web of Science (WOS), and 236 from Scopus. After removing 295 duplicate articles, 556 articles were assessed. Subsequently, we screened for eligibility and inclusion criteria, resulting in the inclusion of 15 papers. The study process and PRISMA flowchart are summarized in Figure 2, while detailed summaries of the included articles can be found in Table 1.

### Quality Assessment and Risk of Bias of Included Articles

**Figure 3:**
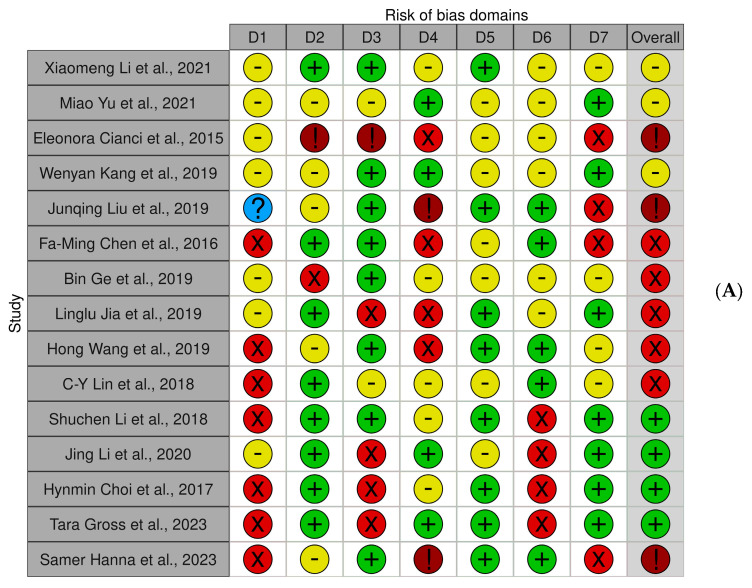

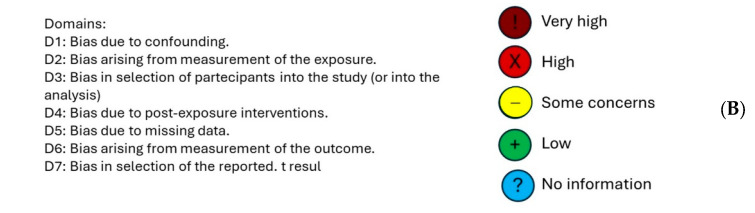
(**A**): Bias assessment; (**B**): legend (Xiaomeng Li et al., 2021 [81], Miao Yu et al., 2021 [82], Eleonora Cianci et al., 2015 [68], Wenyan Kang et al., 2019 [54], Junqing Liu et al., 2019 [83], Fa-Ming Chen et al., 2016 [84], Bin Ge et al., 2019 [85], Linglu Jia et al., 2019 [86], Hong Wang et al., 2019 [87], C-Y Lin et al., 2018 [88], Shuchen Li et al., 2018 [89], Jing Li et al., 2020 [90], Hynmin Choi et al., 2017 [74], Tara Gross et al., 2023 [91], Samer Hanna et al., 2023 [92]).

Figure 3 reports the risk of bias in the included studies. Confounding bias is a major source of bias in most studies. One parameter that has little chance of bias is measurement bias. Due to participant selection bias, there is a modest chance of bias in much research. There is too much heterogeneity to compute the bias resulting from post-exposure. The bias owing to missing data is negligible in much research. There is little bias resulting from outcome measurement. In most studies, there is a significant bias in the selection of the published results. According to the final results, there is a low risk of bias in four studies, a very high risk of bias in three research studies, and a high risk of bias in five studies.

## 4. Discussion

**Figure 4:**
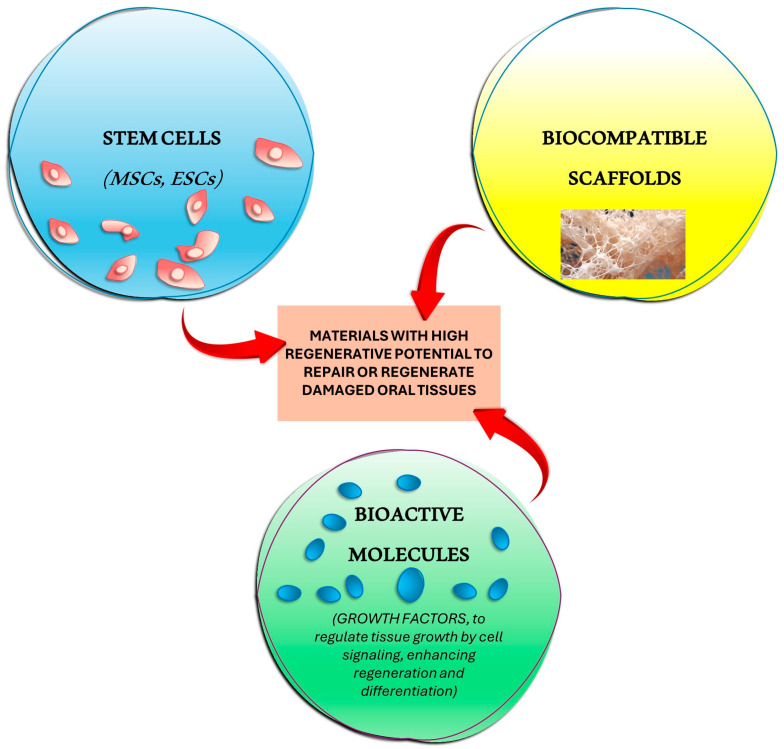
Three key components are the focus of TE: biocompatible scaffolds, stem cells, and bioactive compounds such as drug or growth hormone delivery systems creating materials with strong regenerative potential for the purpose of repairing or regenerating damaged oral tissues in regenerative dentistry.

**Figure 5:**
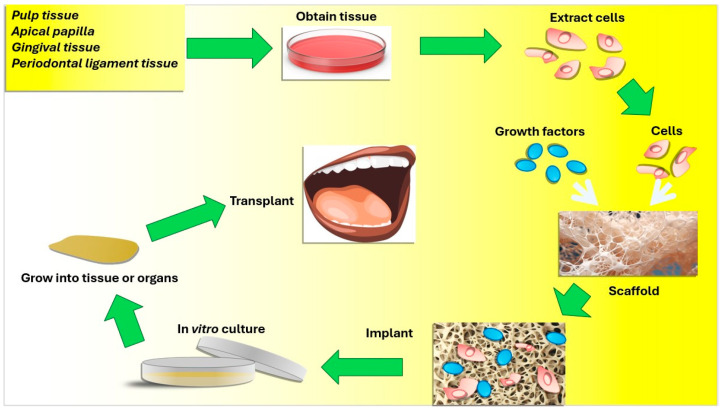
Principles of TE: Various cells extracted from the oral cavity, e.g., pulp tissue, apical papilla, gingival tissue, and periodontal ligament tissue, are seeded on growth factor-soaked scaffolds. The required tissues are obtained after appropriate in vitro culture and finally implanted in vivo.

Recent studies in regenerative medicine have oriented research into new ways of improving the regeneration of dental and bone tissues. Regeneration of dental pulp, periodontal tissue, and alveolar bone represents an innovative procedure that combines tissue engineering and biotechnology to restore vitality to oral tissues (Figure 4 and Figure 5).

### 4.1. Pulp Regeneration

Pulpal regeneration is an innovative dental procedure to give vitality and functionality back to the dental pulp. In order to create new pulp tissue, this strategy combines tissue engineering concepts with biotechnological techniques to maximize the regeneration capacity of stem cells and growth factors. Much research has advanced in the last several years in the fascinating field of using DPSCs to replace lost oral and dental tissues in regenerative treatments [92,93,94,95]. Mitsiadis’ work explored the use of human DPSC and silk fibroin scaffolds for bone regeneration in the craniofacial region. DPSCs were cultured in static and dynamic conditions, with dynamic environments provided by spinner flask bioreactors. Results showed that dynamic conditions, particularly with osteogenic medium, led to significantly higher mineralization and calcium deposition, indicating enhanced bone formation. The study also found that DPSCs can shift away from a dental identity, showing increased expression of bone-related genes under dynamic culture conditions, highlighting their potential for bone tissue engineering [40].

Lin et al. explored the ability of a glycoside of THSG to enhance the self-renewal and expansion of human DPSCs. The study revealed that THSG increased cell viability as well as the rate of plated cells engrafted post-treatment and telomerase activity through modulation of major markers connected to proliferation and stemness. Therefore, THSG can be considered a potential candidate for use in therapeutic rehabilitation in dentistry aimed at preserving the viability of the cells as well as their capacity for regeneration [88,96,97,98].

In a complementary study, Shuchen Li et al. aimed to address the impact of the RNA-binding protein QKI on odontoblastic differentiation of DPSCs [89,90]. Indeed, QKI was shown to promote this specific type of differentiation, which is required for reparative dentinogenesis as a KLF4 ceRNA, by preserving the level of msd mRNA. Thus, the data obtained indicate that QKI could be an important element of the increasing dental structure preservation strategies following dental injuries [99,100,101,102,103].

Tara Gross et al. address in their paper the influence of biomechanical elements, specifically surface elasticity, on DPSC properties. They demonstrated that hard surfaces recruit DPSCs in the creation of hard tissue, whereas soft substrates promote the development of soft tissue using fibronectin-coated polydimethylsiloxane of different stiffness [77,104,105]. This is achieved by changes in focal adhesion proteins, actin cytoskeleton, and cell shape at the level of gene expression. Their research emphasizes how the features of the scaffold material, particularly the elastic moduli, play a crucial role in controlling the destiny of DPSCs, thereby promoting pulp regeneration and mitigating negative consequences [91,106,107,108].

Alongside this, S. Hanna et al. reported that small molecule GSK-3 inhibitors, such as CHIR99021 and tideglusib, promote DPSC expansion by enhancing cell survival via the Wnt/β-catenin pathway through the stabilization of β-catenin. These agents, acting at non-cytotoxic concentrations, are much better alternatives to the conventional growth factors that exploit the potential of regenerative dentistry [92].

These studies, taken together, enhance the regeneration of dental tissues through a variety of means, including biochemical methods, modulation of molecular signaling, biomechanical aspects, and small-molecule treatments. While each study has a particular contribution, they all point out the importance of tailoring therapies in order to manipulate DPSC for effective use in a clinical setting. It is possible that future studies will look at combining such findings in order to enhance existing regenerative treatments in the field of dentistry and oral medicine for the betterment of the patients [96,109,110].

### 4.2. Periodontal Regeneration

The periodontium, which is made up of the alveolar bone, gingiva, periodontal ligament (PDL), and cementum, is essential to both support and maintenance of teeth. Among these, the PDL is especially important for the preservation of periodontal health. Despite this, infections, trauma, orthodontic shifting, or systemic conditions can cause destructive changes in the periodontal tissues, which most often lead to loss of teeth. Restoration of the lost function of the damaged alveolar bone and PDL is also the focus of many of the contemporary medical interventions.

The recent evolution in the field of tissue engineering seeks to utilize every possible stem cell source, with that of periodontal ligament stem cells (PDLSCs) being one of the most promising since they can differentiate into almost every other cell type.

Wenyan Kang et al. give a bright vision about PDLSCs due to their ability for specific tri-lineage differentiation into the osteogenic, adipogenic, and chondrogenic cell lineages, and all these make PDLSCs an excellent candidate for tissue regeneration [111,112,113,114]. However, in spite of such promises, even in Kang’s work mentioned above, practical problems faced by PDLSCs limit their application clinically, such as donor site morbidity, high costs, and contamination risks [115,116,117]. These are remarkable limitations, but in situ tissue engineering, based on exploiting the body’s regenerative potential to attract endogenous stem cells, represents a very promising alternative, though it is also burdened with several complexities [54]. For instance, growth factors like bFGF seem very promising in view of enhancing PDLSCs’ proliferative and differentiative potential, especially when used in combination with BMP-2, which can act synergically at the same time to favor new bone and cementum formation [27,54,118]. Yet, the full potential of such types of approaches remains to be optimized to achieve consistent clinical success [118,119].

In contrast, the article by FaMing Chen et al. (2016) [84] provides a more sober tone in relation to PDLSC treatments in conjunction with Bio-Oss^®^-bovine bone, a product utilized for grafting bones [120]. While the radiographic analysis showed marked filling of bone, clinical improvements in parameters such as CAL, PD, and GR were minimal and statistically insignificant [13,121,122]. This contrast between the visual improvement and clinical outcome underlines the need for further refinement in approaches toward the use of PDLSCs in combination therapies [78,123,124,125,126,127]. It reassures the fact that, though PDLSCs are very promising, methodologies developed to date have not reached the clinical efficacy level to become a standard of care [23,47,117].

Another important aspect explored in this work is the role of PDLSCs in the modulation of inflammation. Eleonora Cianci et al. focus on the role of PDLSCs in controlling the inflammatory response during periodontitis, focusing on their interaction with PMNs, enhancing their antimicrobial function, and preventing their apoptosis. Most importantly, in the work of Cianci, SPMs such as Lipoxin A4 (LXA4) are identified as active players in this process, particularly through the receptor ALX/FPR2, which—except enhancing PDLSC proliferation—also enhances wound healing [68,128,129]. Thus, LXA4 is regarded as a very promising candidate for therapeutic treatment to improve periodontal tissue healing. The results are similarly promising in the study conducted by Miao Yu et al. (2021), which related to the effect of progranulin on PDLSCs [82]. They showed that PGRN not only enhanced ALP activity and the expression of osteogenic genes but also had an anti-inflammatory action against TNF-α, which implies that PGRN can be used as pharmacotherapy targeting periodontal regeneration, especially under inflammatory conditions.

In the study by Xiaomeng Li et al. (2021), surprising results were found: it is a decrease in the levels of prolactin-induced protein (PIP) that actually enhances the osteogenic potential of PDLSCs [81]. This may suggest a regulatory function of PIP in the extracellular matrix, modulating these differentiation processes, which are important in tissue regeneration. Such findings are important to realize the necessity to understand the underlying molecular mechanisms that control PDLSC differentiation and, by manipulating specific proteins like PIP, may lead to the improvement of therapeutic outcomes. Similarly, the work of Bin Ge et al. (2019) on oxytocin (OT) shows that traditionally, lactation and childbirth-associated hormones can act towards the advancement of osteogenic differentiation of PDLSCs and extend the slate of possible therapeutic agents in periodontal tissue engineering [85,130,131,132,133].

Likewise, Miao Yu and others (2021) explored the role of PGRN on osteogenic differentiation of periodontal ligament stem cells (PDLSCs) [134,135].

Their research established that PGRN increased the activity of alkaline phosphatase (ALP) and the expression levels of osteogenic genes, mainly at concentrations of 25 ng/mL, and prevented the inhibitory effects of TNF-α on osteogenic differentiation, thus positioning PGRN as advanced pharmacotherapy for periodontal regeneration in the presence or absence of inflammation [136].

Junqing Liu et al. (2019) continue the study about the influence of inflammatory molecules on stem cell behavior, investigating the action of lipopolysaccharides on stem cells derived from the apical papilla [1,2,3,83]. Significantly, low LPS concentrations promoted osteo/odontogenic differentiation of SCAPs via the MAPK ERK and p38 signaling pathways; therefore, such findings support the hypothesis that controlled exposure to an inflammatory stimulus may prove beneficial for regenerative therapies [1,2,3,83]. It gives a new dimension to the issue of how inflammation interacts with stem cells—a concept that this generally negative inflammatory environment might be employed therapeutically under very controlled conditions [83]. The results suggest that LPS may enhance dental regenerative therapies, but further research and clinical studies are needed to confirm these effects [137,138,139,140,141,142].

Finally, Linglu Jia et al. (2019) present a comparative analysis developed between PDLSCs and GMSCs that reveals significant differences in their biological features. While PDLSCs possess superior differentiating abilities, GMSCs exhibit higher cell proliferation and clonal expansion, thereby making them more suitable for regenerative applications. The investigation goes deep into the genetic underpinnings of such differences, specifying key genes like CDCA7L, PBX1, and PDGFRA overexpressed in GMSCs and contributing to their high proliferative ability [86]. Contrarily, anti-apoptotic genes in PDLSCs may bestow resistance to oxidative stress, thereby giving them some advantage over others in hostile environments, for example, in inflamed periodontal tissues [143,144,145]. This could be an important insight into furthering our understanding of the unique characteristics of these types of stem cells but will also indicate that therapies might be individualized in particular for regenerative needs [33,146].

All these studies detail the possible role of PDLSCs in periodontal regeneration to a high degree. Undeniably, the potential regenerative capability of PDLSCs notwithstanding, much has yet to be agreed upon with respect to practical limitations such as clinical efficacy and consistency in therapeutic outcomes. Another principal attraction of PDLSCs involves their role in the management of inflammation, wherein promising applications appear from their interactions with inflammatory mediators. Conclusively, the findings highlight the fact that deeper insight into the molecular pathways and finer details is required in order to combine PDLSCs either with materials like Bio-Oss^®^ or growth factors like bFGF. Moreover, other molecules, hormones, and even genetic aspects of stem cell types may bring new dimensions to future research and hopefully offer better, more personalized treatments of periodontal diseases.

### 4.3. Bone Regeneration

Discussions, such as those regarding pharmacological interventions, modification of biomaterials, and molecular mechanisms responsible for driving osteogenesis, are fundamental contributions. The study of J. Li et al. about matrine from *Sophora flavescens* Ait. showed its potential to promote bone formation during RME [90]. These studies—including in vitro testing of BMSCs and in vivo rat testing—showed that matrine clearly accelerated the osteogenic differentiation represented by ALP activity and mineralization in BMSCs. It was further revealed that, in the in vivo test, bone volume and density increased due to matrine, which indicates that this compound holds great promise for improving orthodontic-related bone regeneration, such as in RME. However, further extensive studies will be required to fully explain its mechanisms and optimize its clinical applications [89,147,148,149].

While researching, H. Choi et al. also studied the effects of surface treatments on titanium substrates for iPSCs obtained from human gingival fibroblasts [115,150,151,152,153]. They compared machined and SLA-treated surfaces; the latter included sandblasting, large grit, and acid etching. SLA-treated surfaces significantly improved iPSC adhesion, spreading, and osteogenic differentiation [74,154,155,156]. It also increased osteogenic gene expression and mineralization in comparison to machined surfaces. This underlines the importance of surface topography for regulating stem cell behavior and optimizing biomaterials for dental tissue engineering and regenerative medicine applications [74,89,157].

**Figure 6:**
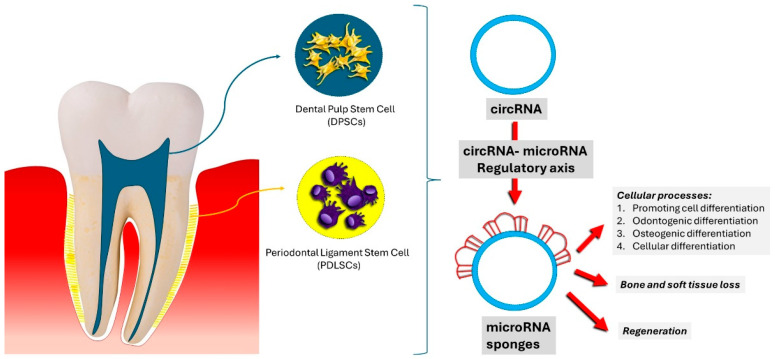
Control of the differentiation of stem cells derived from oral tissues mediated by circRNA.

Adding a layer of complexity, Hong Wang et al. investigated how mechanically induced osteogenic differentiation—which also forms the basis of orthodontic tooth movement—is molecularly controlled by circular RNAs in periodontal ligament stem cells. PDLSCs are indispensable in the process of alveolar bone remodeling, which is dependent on the mechanical forces acting upon it [87,158]. Additionally, circRNAs would act as “sponges” for microRNAs while regulating the expression of genes and thereby influencing cellular differentiation. More than 2900 differently expressed circRNAs could be identified in PDLSCs subjected to mechanical forces in their analysis, indicating that circRNAs represent critical regulators in the cellular response to mechanical stimuli. Thus, the targeted signaling pathways included but were not limited to protein kinase R and ER stress-mediated transcription factor-4, suggesting that circRNAs may be a promising molecular target to enhance bone regeneration for orthodontic treatment (Figure 6) [86,159,160,161,162].

Together, these studies shed light on how different approaches, from the use of pharmacological agents such as matrine to surface modification of biomaterials and molecular regulators, including circRNAs, can all combine toward enhanced osteogenesis and tissue regeneration [74,90,163,164,165]. J. Li et al. presented the use of pharmacological stimulation of osteogenesis in BMSCs, whereas H. Choi et al. emphasized how the surface properties of biomaterials can improve the osteogenic potentials of iPSCs. Hong Wang et al. contributed to the molecular details that underpin how circRNAs mediate responses of stem cells to mechanical forces in bone remodeling [90].

These data indicate that forthcoming studies should combine the pharmacological, biomaterials, and molecular strategies for maximum exploitation of stem cells toward regenerative therapies in both dental and orthopedic applications. This will hopefully allow the development of more effective and selective treatments, improving both clinical practice and the entire field of regenerative medicine.

## 5. Conclusions

Recent advances in dental pulp stem cell (DPSC) research have focused on enhancing stem cell therapies for the regeneration of oral and dental tissues. Researchers have explored various strategies, including biochemical interventions, molecular signaling, biomechanical factors, and small-molecule therapies, to optimize DPSC behavior and boost their therapeutic potential. Key findings include the benefits of substances like 2,3,5,4′-Tetrahydroxystilbene-2-O-β-glucoside (THSG) and GSK-3 inhibitors, which promote DPSC proliferation and viability, aiding tissue regeneration. The role of RNA-binding proteins, such as Quaking (QKI), and biomechanical modulation through different substrate elasticities have been shown to effectively direct DPSC differentiation into specific tissue types, improving pulpal cavitation. Additionally, research on periodontal ligament stem cells (PDLSCs) has yielded promising results in regenerating alveolar tissue and dentinal bone, with growth factors like prolactin and progranulin playing key roles in osteogenic differentiation. PDLSCs also show potential in managing dental inflammation and enhancing tissue elasticity, offering new therapeutic avenues for periodontitis treatment. In the realm of bone regeneration, studies on materials like titanium substrates and surface modifications have highlighted the importance of pharmaceutical interventions and biomaterial properties in promoting osteogenic differentiation and adhesion of induced pluripotent stem cells (iPSCs). These findings suggest that combined approaches, integrating pharmaceutical and biomaterial modifications, could optimize regenerative therapies. Overall, these studies represent significant progress in restorative dentistry, emphasizing the need for integrated approaches to improve stem cell therapies. Future research should continue exploring these avenues to enhance clinical outcomes and patient quality of life in oral medicine and dentistry.

## Figures and Tables

**Table 1:** Qualitative analysis of the studies included.

Authors (Year)	Type of the Study	Aim of the Study	Materials	Results
Xiaomeng Li et al., 2021 [81]	Experimental study	Investigate mechanisms of osteogenic differentiation in periodontal ligament stem cells (PDLSCs), focusing on prolactin-induced protein (PIP) expression.	Used PDLSCs, employed short-airpin Ribo Nucleic Acid (shRNA) to reduce PIP expression, and analyzed effects on proliferation, apoptosis, and osteogenic differentiation.	Reduced PIP enhanced osteogenic differentiation of PDLSCs without affecting proliferation or apoptosis, suggesting a regulatory role of PIP in extracellular matrix (ECM) management during differentiation.
Miao Yu et al., 2021 [82]	Clinical trial	Evaluate the impact of progranulin (PGRN) on osteogenic differentiation of PDLSCs and its interaction with TNF-α.	Treated PDLSCs with varying concentrations of PGRN assessed alkaline phosphatase (ALP) activity, gene expression (ALP, Runx2), and ECM mineralization.	Optimal PGRN concentration (25 ng/mL) promoted osteogenic differentiation and counteracted TNF-α inhibition, enhancing ALP activity and ECM mineralization.
Eleonora Cianci et al., 2015 [68]	Experimental study	Investigate human PDLSCs’ role in inflammation resolution in periodontitis, focusing on specialized pro-resolving lipid mediators (SPMs) like lipoxin A4 (LXA4).	Explored interactions between PDLSCs and polymorphonuclear neutrophils (PMNs), evaluated SPM synthesis, and effects of LXA4 on cell functions.	Human PDLSCs enhance PMN functions and produce SPMs (LXA4, RvDs, maresins), with LXA4 enhancing PDLSC proliferation, migration, and wound healing.
Wenyan Kang et al., 2019 [54]	Experimental study	Discuss challenges and strategies in periodontal therapy using PDLSCs, focusing on in situ tissue engineering and growth factors (bFGF, transplant-2).	Reviewed literature on PDLSC-based therapies discussed in situ tissue engineering and growth factor applications.	Sequential application of bFGF and BMP-2 enhanced PDLSC proliferation, migration, and osteogenic differentiation, promoting new bone and cementum tissue formation.
Junqing Liu et al., 2019 [83]	Experimental study	Investigate lipopolysaccharide (LPS) influence on cells from the apical papilla (SCAPs) differentiation into bone and dental tissues.	Exposed SCAPs to varying LPS concentrations assessed osteo/odontogenic differentiation and mitogen-activated protein kinase (MAPK) signaling pathways.	LPS at 0.1 μg/mL enhanced SCAPs’ osteo/odontogenic differentiation via MAPK ERK and p38 pathways, suggesting potential for enhancing dental regenerative therapies.
Fa-Ming Chen et al., 2016 [84]	Clinical trial	Evaluate the efficacy of PDLSCs combined with Bio-Oss^®^ for periodontitis treatment.	Conducted a randomized clinical trial that assessed bone height and clinical parameters post-treatment.	PDLSCs with Bio-Oss^®^ showed significant bone fill, confirming safety and potential efficacy, though there were no significant differences compared to controls in clinical parameters.
Bin Ge et al., 2019 [85]	Experimental study	Examine oxytocin (OT) role in PDL regeneration and PDLSC osteogenic differentiation.	Tested OT effects on PDLSC proliferation, migration, and osteogenic differentiation and analyzed signaling pathways (MAPK/ERK, AKT).	OT enhanced PDLSCs’ proliferation, migration, and osteogenic differentiation, mediated through MAPK/ERK and AKT pathways, suggesting potential for periodontal regeneration.
Linglu Jia et al., 2019 [86]	Experimental study	Compare the biological characteristics and gene expression profiles of PDLSCs and gingival-derived mesenchymal stem cells (GMSCs).	Analyzed mRNA and lncRNA expression and compared differentiation capabilities and proliferation rates between PDLSCs and GMSCs.	PDLSCs showed superior osteogenic, adipogenic, and chondrogenic differentiation; GMSCs exhibited higher proliferation rates and unique gene expression profiles, highlighting potential applications in regenerative medicine.
Hong Wang et al., 2019 [87]	Experimental study	Investigate circular RNAs (circRNAs) role in PDLSCs during mechanically induced osteogenic differentiation.	Examined circRNA expression in PDLSCs under mechanical forces and analyzed pathways (protein kinase R, ER stress-mediated transcription factor-4).	Identified over 2900 differentially expressed circRNAs in mechanically stimulated PDLSCs, suggesting circRNAs’ potential role in bone regeneration and orthodontic therapies.
C-Y Lin et al., 2018 [88]	Experimental study in vitro.	To investigate the effect of 2,3,5,4′-Tetrahydroxystilbene-2-O-β-glucoside (THSG) on human dental pulp stem cells (hDPSCs) and the mechanisms enhancing its proliferative potential.	Cell viability assays, mRNA expression analysis, flow cytometry, Western blotting.	THSG increases cell viability, colony formation, and telomerase activity in hDPSCs, suggesting potential applications in dental regeneration.
Shuchen Li et al., 2018 [89]	In vitro, in vivo	To investigate how hDPSCs differentiate into odontoblasts and the function of the RNA-binding protein Quaking (QKI) in this process.	Cultures of hDPSCs	QKI acts as a competing endogenous RNA (ceRNA), promoting odontoblastic differentiation.
Jing Li et al., 2020 [90]	In vitro and in vivo	Evaluate matrine’s effect on bone formation during RME.	In vitro: Used rat bone marrow mesenchymal stem cells (BMSCs) to test matrine’s osteogenic effect through ALP activity, mineralization, and osteogenic markers. In vivo: Rats underwent RME with and without matrine, followed by micro-CT and histological analysis.	Matrine enhanced BMSCs osteogenic differentiation in vitro and improved bone density, trabecular number, and thickness in vivo during RME. Suggests matrine as a potential therapy for enhancing bone formation and stability in orthodontics and maxillofacial surgery.
Hynmin Choi et al., 2017 [74]	Experimental	Investigating the behavior of induced pluripotent stem cells (iPSCs) on titanium surfaces with different textures.	To investigate the behavior of iPSC pluripotent stem cells derived from human gingival fibroblasts, they were cultured on sandblasted with large grit and acid-etched titanium surfaces.	iPSCs show improved initial adhesion, diffusion, osteogenic gene expression, and mineralization on sandblasted and acid-etched (SLA-treated) surfaces compared to processed surfaces.
Tara Gross et al., 2023 [91]	Empirical Study	To investigate whether dental pulp stem cells (DPSCs) can be directed toward soft tissue differentiation by extracellular elasticity.	Enriched STRO-1-positive DPSCs cultured on substrates with 1.5, 15, and 28 kPa elasticities. Gene transcription via qPCR.	1.5 kPa led to soft tissue phenotype. 28 kPa showed hard tissue differentiation. 15 kPa had the highest cytokine expression. Biophysical cues significantly impact DPSC fate.
Samer Hanna et al., 2023 [92]	In vitro Study	Investigate CHIR99021 and tideglusib’s effects on hDPSCs’ proliferation, viability, and stemness.	- Isolation of hDPSCs from premolars extracted for orthodontic purposes. - Assessment of cytotoxicity and proliferation using cell counting kit-8 and flow cytometric analysis of ANNEXIN V. - Analysis of stemness markers via quantitative real-time polymerase chain reaction (qRT-PCR). - Evaluation of tideglusib (100 nM) and CHIR99021 (5 nM) concentrations on cell viability and proliferation.	Both compounds safely promoted hDPSC proliferation, minimized apoptosis (low ANNEXIN V), and boosted stemness marker expression, suggesting their potential for regenerative dentistry.

## Data Availability

Data sharing is not applicable to this article as no new data were created or analyzed in this study.

## Abbreviations

The following abbreviations are used in this manuscript.

AKT	Protein kinase B
ALP	Alkaline phosphatase
ANNEXIN V	apoptotic marker
bFGF	Basic fibroblast growth factor
BMP-2	Bone morphogenetic protein 2
BMSCs	Bone marrow mesenchymal stem cells
ceRNA	Competing endogenous RNA
circRNA	Circular RNA
CDCA7L	Gene name
CCL2	Gene name
DPSC	Dental pulp stem cell
ECM	Extracellular matrix
ERAP2	Gene name
ERK	Extracellular signal-regulated kinase
GMSCs	Gingival-derived mesenchymal stem cells
GSK-3	Name of the molecule
hDPSC	Human dental pulp stem cells
IPScs	Induced pluripotent stem cells
LPS	Lipopolysaccharide
LXA4	Lipoxin A4
mRNAs	Messenger RNAs
MAPK	Mitogen-activated protein kinase
MSC	Mesenchymal stem cells
OT	Oxytocin
PDL	Periodontal ligament
PDLSCs	Periodontal ligament stem cells
PGD2, E2, F2a	Prostaglandins D2, E2, F2a
PGRN	Progranulin
PIP	Prolactin-induced protein
PMN	Polymorphonuclear neutrophils
PRDX5, PRDX6, TXN2	Gene names
Qpcr	Quantitative polymerase chain reaction
qRT-PCR	Quantitative real-time polymerase chain reaction
QKI	Quaking
RME	Rapid maxillary expansion
RNA	Ribonucleic acid
Runx2	Runt-related transcription factor 2
SCAPs	Stem cells from the apical papilla
shRNA	Short airpin RNA
SLA	Sandblasted and acid etched
SPMs	Specialized pro-resolving lipid mediators
THSG	2,3,5,4′-tetrahydroxystilbene-2-O-β-glucoside
TNF-α	Tumor necrosis factor alpha
